# Nanogels Containing *Foeniculum vulgare* Mill. and *Mentha piperita* L. Essential Oils: Mosquitoes' Repellent Activity and Antibacterial Effect

**DOI:** 10.1155/2022/4510182

**Published:** 2022-08-31

**Authors:** Alireza Sanei-Dehkordi, Abbas Abdollahi, Mojdeh Safari, Farnaz Karami, Ghazal Ghaznavi, Mahmoud Osanloo

**Affiliations:** ^1^Department of Medical Entomology and Vector Control, School of Health, Hormozgan University of Medical Sciences, Bandar Abbas, Iran; ^2^Infectious and Tropical Diseases Research Center, Hormozgan Health Institute, Hormozgan University of Medical Sciences, Bandar Abbas, Iran; ^3^Department of Microbiology, School of Medicine, Fasa University of Medical Sciences, Fasa, Iran; ^4^Department of Medical Nanotechnology, School of Advanced Technologies in Medicine, Tehran University of Medical Sciences, Tehran, Iran; ^5^Department of Medical Biotechnology, School of Advanced Technologies in Medicine, Fasa University of Medical Sciences, Fasa, Iran; ^6^Department of Medical Nanotechnology, School of Advanced Technologies in Medicine, Fasa University of Medical Sciences, Fasa, Iran; ^7^Noncommunicable Disease Research Center, Fasa University of Medical Sciences, Fasa, Iran

## Abstract

*Foeniculum vulgare* Mill. and *Mentha piperita* L. are two common medicinally important plants with a wide range of biological activities such as insecticide and antibacterial effects. In this study, the chemical composition of their essential oils was investigated using GC-MS analysis. After that, their nanoemulsions were prepared; optimum samples with droplet sizes of 74 ± 7 and 136 ± 5 nm were gelified. The viscosity of the prepared nanogels and the successful loading of the essential oil in them were investigated. The efficacy of the nanogel containing *M. piperita* essential oil as a repellent and antibacterial agent was more potent than the nanogel containing *F. vulgare* essential oil. Its completely protected time against *Anopheles stephensi*, the main malaria mosquito vector, was 120 ± 8 min. Moreover, the growth of *Escherichia coli* and *Staphylococcus aureus* after treatment with 5000 µg/mL of nanogel containing *M. piperita* essential oil was reduced by 100 and 65%, respectively. Considering natural constituents, a straightforward preparation method, and high efficacy, the nanogel containing *M. piperita* essential oil could be introduced for further investigation against other mosquitoes and bacterial species.

## 1. Introduction

Essential oils are volatile natural oils formed as secondary metabolites in aromatic plants [1]. They are extracted from different parts of plant organs, e.g., buds, flowers, leaves, stems, twigs, seeds, fruits, roots, wood, or bark [2]. They have many biological effects, such as antiseptic, antibacterial, antiviral, and fungicidal properties [3, 4]. Besides, their larvicidal activity, repellent effects, and insecticide properties have also been confirmed [5, 6]. However, they should be stabilized due to their volatility and instability [7]. Preparation of EO-based nanoformulation has been recently considered a promising approach [8]. Among the common nanoformulations such as nanoemulsions, polymeric nanoparticles, and lipid nanocarriers, nanogels have received much attention, especially in topical applications, due to proper viscosity, high loading capacity, biocompatibility, and biodegradability [9, 10].

*Escherichia coli* and *Staphylococcus aureus* are two Gram-negative and positive opportunistic pathogens that (could) cause severe and life-threatening human infections [11]. *S. aureus* is mainly responsible for postoperative wound infection, toxic shock syndrome, and food poisoning [12]. The *E. coli* is present in the human intestine and causes lower urinary tract infection, coleocystis, or septicemia [13]. These bacteria enter the body through contaminated hands and can, of course, cause skin damage. Moreover, *S. aureus* is an important cause of soft tissue and skin infections such as boils, impetigo, carbuncles, staphylococcal scalded skin syndrome, and cellulitis.

Furthermore, malaria, with about 241 million cases and 627,000 deaths in 2020, is still the most dreadful of mosquito-borne diseases [14]. Around 30 species of the 400 identified *Anopheles* mosquito species are the vectors of malaria to humans [15]. *Anopheles stephensi* Liston is one of the most important malaria vectors in the Middle East and South Asia [16, 17]. However, it has recently expanded to Ethiopia, Djibouti, Lakshadweep, and Sri Lanka [18]. Besides, mosquito bites can be infected by other pathogens such as bacteria. However, one recommended solution to prevent mosquito-borne disease transmission is to use repellents [19, 20]. On the other hand, resistance to industrial repellents and their adverse effects has been one of the challenges for health systems in recent years [21, 22]. For instance, DEET (N, N-diethyl-3 methylbenzamide) is one of the best known and most successful synthetic chemical repellents that, by blocking the olfactory receptors neurons, causes repellency [23, 24]. However, its usage has been questioned due to its side effects on humans such as hypotension, seizures, neurotoxic, and skin irritations [25, 26].

*Foeniculum vulgare* Mill. and *Mentha piperita* L. are two common medicinally important plants with a wide range of biological activities such as antibacterial and insecticide effects [27, 28]. For instance, the literature reported that *F. vulgare* EO at a concentration of 40 mg/L caused 50% mortality for the second instars larvae *Culex pipiens* [29]. Besides, a protection time of 0.5% *M. piperita* EO against *An. stephensi* was reported at 17 min [30].

This study was an attempt to prepare multifunctional topically administrated natural nanogels. First, two nanogels containing *F. vulgare* and *M. piperita* EOs were prepared. After that, their antibacterial activities against *E. coli* and *S. aureus* were investigated. Finally, their repellent efficacies were investigated against *An. stephesni* compared to DEET as a gold standard repellent.

## 2. Materials and Methods

### 2.1. Materials

*F. vulgare* and *M. piperita* EOs were bought from Tabib Daru Company (Iran) and Zardband Pharmaceuticals Company (Iran). *S. aureus* (ATCC 25923) and *E. coli* (ATCC 25922) were provided by the Pasteur Institute of Iran. Carboxymethylcellulose (CMC), Mueller–Hinton broth, Mueller–Hinton agar, and Tween 20 were bought from Merck Chemicals (Germany). DEET 40% was purchased from Reyhan Naghsh Jahan Pharmaceutical Co., Iran. It was diluted to 2.0% using distilled water as the diluent following the formulated nanogel products.

### 2.2. Chemical Composition of the EOs

A gas chromatography device (Agilent 6890, HP-5MS column, USA) connected to a mass spectrometer (Agilent 5973, USA) was used for chemical compositions of the EOs as described in our previous study. Besides, relative abundances were calculated by peak area normalization [31].

### 2.3. Preparation and Characterization of Nanoemulsion-Based Gels

A fixed amount of (2.0% v/v) each EO was mixed with different amounts of Tween 20 (2000 rpm, 3 min, room temperature) to form a homogenous mixture. Distilled water was then added dropwise up to the final volume (5000 µL) and stirred for 40 minutes. The prepared nanoemulsions were subjected to size analysis using a DLS-type apparatus (K-One Nano Ltd., Korea). Nanoemulsions with proper size characteristics, including droplet size of <200 nm and droplet size distribution (SPAN) less than 1 [32], were considered optimum samples.

**Figure 1:**
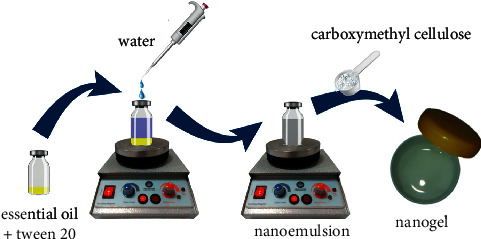
Preparation of nanoemulsion-based nanogels.

An optimum nanoemulsion from each EO (No. 1 and No. 7,Table 1) was selected for gelation; CMC (3.5% w/v) was added to each and stirred (2000 rpm) overnight at room temperature to complete the gelation process. The prepared nanogels containing *F. vulgare* and *M. piperita* EO were abbreviated as FVNG and MPNG. A schematic of the described method is depicted in Figure 1. Furthermore, blank gels of each nanogel were also prepared using the same approach, only without EO.

The viscosity of FVNG and MPNG at shear rates of 0.1–100 1/s was investigated using a Rheometer machine (MCR-302, Anton Paar, Austria). Moreover, ATR-FTIR analysis was used to investigate the successful loading of the EOs in the nanogels; spectra of EOs, blank gels, and nanogels (FVNG and MPNG) were recorded in a wavenumber range of 400–3900 cm^−1^. Without any preparation process, the samples were subjected to the spectrometer apparatus (Tensor II model, Bruker Co, Germany).

### 2.4. Repellent Bioassay

Susceptible mosquitoes (Bandar-e-Abbas strain) were used in the current study. They were reared and maintained at 27 ± 2 C temperature, ≥70 ± 10% relative humidity, and a 12 : 12 (light: dark) photoperiod. For repellent bioassays, 250 nonblood fed and nulliparous 5–7 days old adult female mosquitoes were kept in cages (40 × 40 × 40 cm) and not fed for 14 h before repellency tests. A 47-year-old male volunteer was employed to determine the protection time using the Arm-in-cage method with a slight modification [33]. His forearm was first washed with 70% alcohol and dried with a towel. Only the underside of the lower arm between the wrist and elbow, with an area of 8 cm × 12.5 cm (covered by fewer hairs), was exposed, and latex gloves covered the hand. The volunteer's hands were then impregnated with the samples (1g of FVNG and MPNG, 1 mL of DEET). After 5 minutes, the volunteer placed his forearm in a cage for 3 minutes. This procedure is repeated at a 30-minute interval; the test was stopped when one landing and/or probing occurred in a 3 min test.

### 2.5. Antibacterial Tests

For investigation of antibacterial tests of FVNG and MPNG as well as their blank gels, the ATCC100 assay was used with slight modifications [32]. Four mL of each fresh bacterial suspension (2 × 10^5^ CFU/mL) was first filled separately in 6 cm plate dishes. Then, by adding 1.0, 0.5, and 0.25 g of each nanogel, final concentrations of EOs were fixed at 5000, 2500, and 1250 µg/mL. Antibacterial effects of blank gels were also investigated in a similar process. The treated plates were then incubated at 37°C for 24 h, and 10 µL of suspensions was cultured on a Muller–Hinton agar culture plate and incubated for 24 h. Finally, the number of grown colonies was counted and compared with the control group, and growth reduction was calculated using the following equation.

(1) $$\begin{array}{c} \text{Bacterial growth }\left(\text{\%}\right)=\left(\frac{\text{CFU sample}}{\text{CFU control}}\right)\times 100\text{.} \end{array}$$

### 2.6. Statistical Analyses

All experiments were repeated three times, and the results were given as mean ± standard deviation. For the comparison of two or higher samples, the independent sample *t*-test and one-way ANOVA with at least a 0.05 significance level were used (STATA v11, StataCorp, USA).

## 3. Results

### 3.1. Compounds of *F. vulgare* and *M. piperita* EOs

Identified compounds in the EOs using GC-MS analysis are listed in Table 2. *trans*-Anethole (52.6%), limonene (11.1%), carvone (8.2%), tarragon (7.7%), and fenchone (4.5%) are five major compounds in *F. vulgare* EO. Besides, menthol (31.1%), menthone (22.1%), camphane (7.0%), menthofuran (6.0%), and iso-menthone (5.9%) are major compounds in *M. piperita* EO.

### 3.2. Ten Prepared Nanoemulsions and Two Nanoemulsion-Based Nanogels

**Figure 2:**
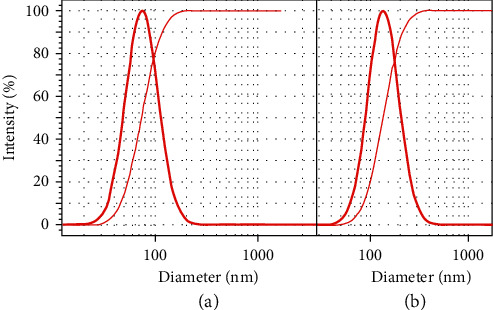
DLS profiles of optimum nanoemulsions containing EOs of (a) *F. vulgare* (74 ± 7 nm) and (b) *M. piperita* (136 ± 5 nm).

Five nanoemulsions containing 2.0% *F. vulgare* EO were prepared (Table 2, Nos. 1–5). Sample No. 1, with proper size characteristics, i.e., droplet size of 74 ± 7 nm and SPAN of 0.97, was selected as the optimum sample for gelation. In addition, five nanoemulsions containing *M. piperita* EO (Table 2, Nos. 6–10) were prepared; No. 7 with 136 ± 5 nm droplet size and 0.96 SPAN was selected as the optimum nanoemulsion for gelation. DLS profiles of two selected nanoemulsions are depicted in Figure 2.

**Figure 3:**
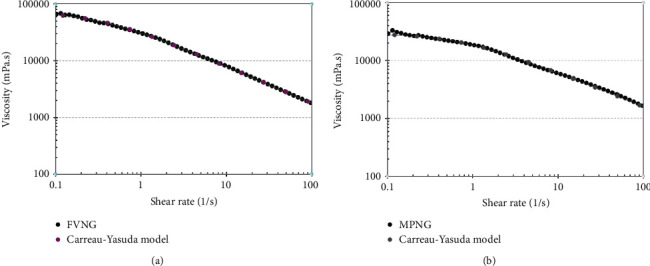
Viscosity of the nanogels containing (a) *F. vulgare* EO (FVNG) and (b) *M. piperita* EO (MPNG) at different shear rates fully fitted with the Carreau–Yasuda model.

Two of the mentioned nanoemulsions were gelified using 3.5% w/v CMC. Their viscosity at different shear rates (0.1–100 1/s) was fully fitted with a well-known non-Newtonian regression, the Carreau–Yasuda model (Figure 3). In non-Newtonian fluids, viscosity decreases with an increasing shear rate [34].

### 3.3. Successful Loading of the EOs in Nanogels

**Figure 4:**
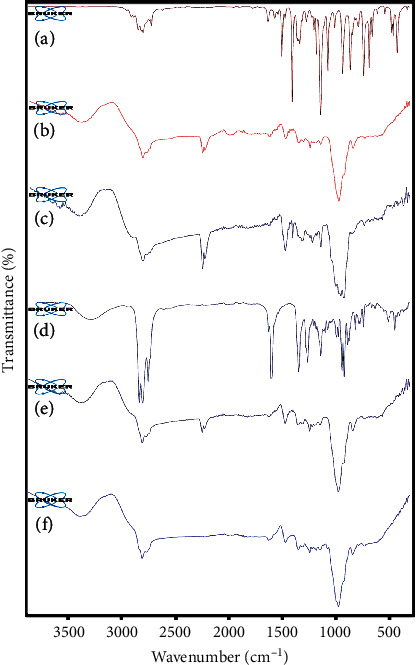
ATR-FTIR spectra of (a) *F. vulgare* EO, (b) blank gel, (c) nanogel containing *F. vulgare* EO (FVNG), (d) *M. piperita* EO, (e) blank gel, and (f) nanogel containing *M. piperita* EO (MPNG).

In the ATR-FTIR spectrum of *F. vulgare* EO (Figure 4(a)), the absorption peaks at around 2834–3022 cm^−1^ are associated with stretching –OH and NH_2_ groups. The peaks observed at 1737 cm^−1^ and 1607 cm^−1^ are assigned to the stretching vibration of the –C=O group. Several characteristic peaks appeared at 1509 cm^−1^ (-NH is plane bend and –CN stretching), 1414, 1440, and 1243 cm^−1^ (-NH bending and –CN stretching), 1035–1174 cm^−1^ (aromatic C–H in the plane bend), and 837 cm^−1^ (out of the plane –NH bending). A similar observation for *F. vulgare* EO was reported in the literature [35]. In the spectra, blank gel (Figure 4(b)), the prominent peak at 3508–3698 cm^−1^ is assigned to the OH group in CMC [36]. The bands that appeared at 2922, 1581, and 1461 cm^−1^ are related to the stretching vibration of C-H, C=O stretching, and hydrocarbon groups (-CH_2_), respectively, in CMC. The peak at 1080–1252 cm^−1^ is attributed to the CMC's ether groups (-O- stretching). In *F. vulgare*, nanogel (FVNG) (Figure 4(c)) retained most of the peaks that appeared in the spectra of blank gel and *F. vulgare* EO confirmed the loading of the EO into the nanogel structure, although some changes in the position and intensity of peaks were identified.

In the spectrum of *M. piperita* (Figure 4(d)), different functional groups such as alkanes, phenols, alkenes, ethers, alcohol, ester, and carboxylic acid are observed [37, 38]. The major peak at 3400 cm^−1^ is attributed to the hydrogen-bonded alcohol and phenols. The bands observed at 2869, 2922, 2953, 1287, 1368, and 1455 cm^−1^ are assigned to alkanes' C–H stretching. The absorption peak observed at 1710 cm^−1^ is related to C=C starching. The bands that appeared at 1044 and 1079 cm^−1^ are attributed to the C–O vibration of ethers, alcohol, esters, and carboxylic acids. The characteristic band around 875 cm^−1^ could be related to alkenes' C–H bonds. The main absorption peaks of blank gel (Figure 4(e)) of this nanogel are like previous blank gel and interpreted above. In the FTIR spectrum of *M. piperita* EO nanogel (Figure 4(f)), some peaks' changes in shape and intensity were identified, which correspond to the possible interaction between *M. piperita* EO and CMC. For instance, the absorption band at 1581 cm^−1^ attributed to C=O in CMC was shifted to 1579 cm^−1^ in the spectrum of prepared nanogel. Moreover, the intensity of the peaks observed at 2869, 2922, and 2953 cm^−1^ assigned to the C–H stretching of alkanes in *M. piperita* EO was changed in the spectrum of the final nanogel. Finally, the presence of characteristic peaks of both CMC and *M. piperita* EO in the FTIR spectra of MPNG indicates both components' existence in the structure of obtained nanogels.

### 3.4. Repellent Properties of the Nanogels

**Figure 5:**
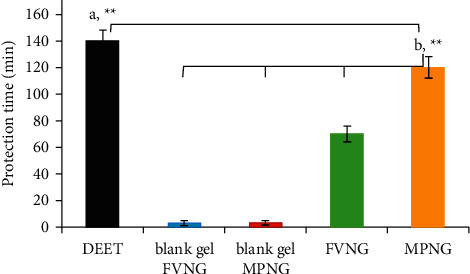
Complete protection times of nanogels containing *F. vulgare* and *M. piperita* EOs (FVNG and MPNG), blank gels (without EO), and DEET against *An. stephensi*. (a) Efficacy of DEET is more than MPNG (^*∗∗*^*P* < 0.01). (b) The efficacy of MPNG is more potent than FVNG and both blank gel (^*∗∗∗*^*P* < 0.001).

From Figure 5, MPNG, with a complete protection time of 120 ± 8 min, was significantly more potent than FVNG (70 ± 6). However, its efficacy was less than DEET, with a protection time of 140 ± 8 (*P* < 0.01). Besides, both blank gels with 3 min of complete protection time did not show proper efficacy.

### 3.5. Antibacterial Effects of the Nanogels

**Figure 6:**
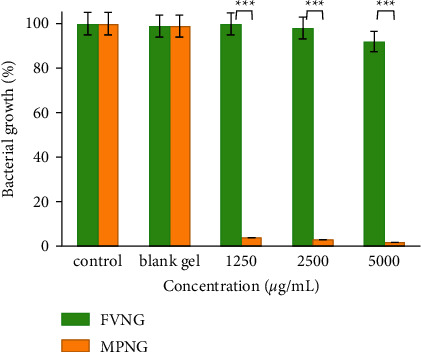
Antibacterial effects of nanogels containing *F. vulgare* and *M. piperita* EOs (FVNG and MPNG) and their blank gels (without EO) against *E. coli*. The efficacy of MPNG was more potent (^*∗∗∗*^*P* < 0.001) than FVNG.

**Figure 7:**
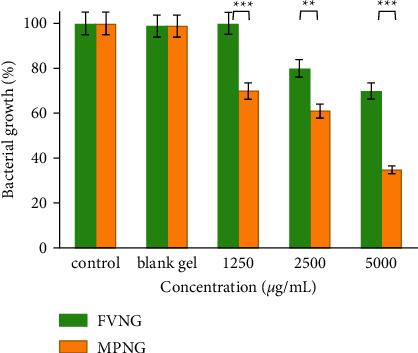
Antibacterial effects of nanogels containing *F. vulgare* and *M. piperita* EOs (FVNG and MPNG) and their blank gels (without EO) against *S. aureus*. The efficacy of MPNG was more potent (^*∗∗∗*^*P* < 0.001 and ^*∗∗*^*P* < 0.01) than FVNG.

The antibacterial effects of the nanogels and their blank gels against *E. coli* and *S. aureus* are depicted in Figures 6 and 7. The growth of *E. coli* after treatment with 1250, 2500, and 5000 µg/mL of FVNG was not significantly reduced (growth ≥ 92%). However, the growth of *S. aureus* after treatment with 5000 µg/mL FVNG was reduced by 30%.

Besides, after treatment with MPNG 1250, 2500, and 5000 µg/mL, the growth of *E. coli* was substantially reduced (≥ 96%). However, after treating *S. aureus* at those concentrations, bacterial growth was observed at 70, 61, and 35%. Moreover, both nanogels did not affect the growth of both bacterial types.

## 4. Discussion

Mosquitoes (*Diptera*: *Culicidae*) transmit malaria, dengue, yellow fever, encephalitis, filariasis, chikungunya, and Zika virus [39, 40]. Indoor residual spraying and insecticide-impregnated bed nets are core components of malaria prevention and elimination strategies, and repellents are also recommended in endemic regions [41, 42]. Repellents are substances that deter mosquitoes (or insects) from flying to, landing on, or biting human, animal skin, and surfaces [43, 44]. Due to mosquitoes' resistance to industrial repellents and their adverse effects on human health, many attempts have recently been made to develop natural nanorepellents. For instance, a solid lipid nanoparticle containing 1% *Zataria multiflora* EO was introduced with a 90 min protection time against *An. stephensi* [45]. Besides, the literature reported nanoemulsions containing 15% *Eucalyptus globulus* EO with 170 min protection time against a mixture of mosquitoes [46]. Moreover, nanoemulsion containing 50% *M. piperita* EO with 257 min protection time against *An. stephensi* was also reported [47].

In developing essential oil-based repellents, controlling the pungent odor of EOs is a challenge; the corresponding author of this article has observed that volunteers refuse to use pungent odor repellents. A practical solution to control the odor of colloidal nanoformulations (such as nanoemulsion or nanoparticles) is to turn them into nanogels. Besides, their topical application is also facilitated due to the increased viscosity. Therefore, in the current study, nanogels dosage form was used; interestingly, MPNG showed a 120 min repellent effect against *An. stephensi*.

Symbiotic and opportunistic bacteria commonly influence the skin as the body's first barrier against environmental pathogens; they can cause pain, swelling, and skin color changes [48, 49]. Moreover, some opportunistic bacteria could enter the body through open wounds, possibly leading to bloodstream infections like septicemia [50, 51]. In the current study, the growth of *E. coli* after treatment with 1250 µg/mL MPNG was reduced by more than 95%. On the other hand, the efficacy of MPNG against *S. aureus* was less than *E. coli*; the growth after treatment with 5000 µg/mL was reduced by 65%. Some reports with promising antibacterial effects of nonformulated EOs and nanostructures containing EOs have been found in the literature. For instance, the antibacterial activity of *Juniper communis* EO against standard strains of *Staphylococcus aureus*, *Escherichia coli*, *Pseudomonas aeruginosa*, and *Streptococcus pyogenes* was investigated [52]. Besides, the viability of *S. aureus* and *E. coli* after 24 h exposure to polycaprolactone nanofibers containing *Mentha piperita* EO was decreased to around 50% [53]. Moreover, the *E. coli* colonies were reduced by about 4.0 log CFU/mL after treatment with thyme EO nanoemulsion [54].

## 5. Conclusions

This study aimed to develop two natural nanogels using *F. vulgare* and *M. piperita* EOs as mosquito repellent and antibacterial agent prototypes. The complete protection times of the nanogels against *An. stephensi* were observed as 70 (±6) and 120 (±8) min. The nanogel containing *F. vulgare* EO showed some degree of antibacterial effects against *E. coli* and *S. aureus*. However, after treatment with nanogel of *M. piperita* EO 100 and 65%, their growth was reduced. Therefore, the nanogel of *M. piperita* EO could be considered for further investigations as mosquito repellent and antibacterial agent.

## Figures and Tables

**Table 1:** Prepared nanoemulsions and their size analyses.

No.	*F. vulgare* (%)	*M. piperita* (%)	Tween 20 (%)	Droplet size (nm)	SPAN^a^
1	2	—	3	74	0.97
2	2	—	4	45	3.6
3	2	—	6	27	7.4
4	2	—	8	12	5.4
5	2	—	10	132	2.7
6	—	2	3	76	5.1
7	—	2	4	136	0.96
8	—	2	6	7	1.5
9	—	2	8	22	3.2
10	—	2	10	395	1.8

^a^Droplet size distribution.

**Table 2:** Identified compounds in the EOs using GC-MS analysis.

RT^a^	Compound	*F. vulgare*			*M. piperita*		
		Area	%	RI^b^	Area	%	RI
7.0	*α*-Pinene	2358139618	1.4	932	—	—	—
8.3	*β*-Pinene	—	—	—	63432312	1.3	979
9.5	*α*-Phellandrene	1929797231	1.2	1027	—	—	—
11.1	Limonene	18097975121	11.1	1029	—	—	—
13.4	Fenchone	7258724167	4.5	1083	—	—	—
13.9	1,8-Cineole	—	—	—	207426444	4.1	1026
15.7	*trans*-Sabinene hydrate	—	—	—	47072634	1.0	1098
18.1	Tarragon	12583158254	7.7	1196	—	—	—
19.6	Fenchyl acetate	4922665422	3.0	1218	—	—	—
20.0	Carvone	13304259306	8.2	1243	—	—	—
20.0	Menthone	—	—	—	1105246066	22.1	1152
20.3	Iso-menthone	—	—	—	293121099	5.9	1162
20.4	Menthofuran	—	—	—	301336044	6.0	1164
21.2	Menthol	—	—	—	1553773516	31.1	1172
23.5	Pulegone	—	—	--	104297121	2.1	1273
23.8	*trans*-Anethole	85700289131	52.6	1284	—	—	—
26.2	Camphane	—	—	—	351577121	7.0	1131
31.5	*trans*-Caryophyllene	—	—	—	150233448	3.0	1419
34.0	Germacrene D	—	—	—	92534749	1.9	1481
35.6	Dillapiole	1547681987	1.0	1622	—	—	—

^a^Retention time (min), ^b^retention index.

## Data Availability

The data used to support this study are available from the corresponding author upon request.
